# Proteomic analysis identifying proteins relevant for treatment success following experimental neonatal inflammation-sensitized hypoxia-ischemia

**DOI:** 10.1038/s41390-025-04097-8

**Published:** 2025-05-13

**Authors:** Hannah Burkard, Damjan Osredkar, Elke Maes, Maria E. Bernis, Anna-Sophie Bremer, Margit Zweyer, Paul Dowling, Kay Ohlendieck, Marianne Thoresen, Hemmen Sabir

**Affiliations:** 1 Department of Neonatology and Pediatric Intensive Care, Children’s Hospital, University of Bonn, 53127 Bonn, Germany; 2 Deutsches Zentrum für Neurodegenerative Erkrankungen (DZNE), 53127 Bonn, Germany; 3 Department of Pediatric Neurology, University Children’s Hospital, University Medical Centre Ljubljana, Ljubljana, Slovenia; 4 Faculty of Medicine, University of Ljubljana, Ljubljana, Slovenia; 5 Department of Biology, Maynooth University, National University of Ireland, W23 F2H6 Maynooth, Co, Kildare, Ireland; 6 Kathleen Lonsdale Institute for Human Health Research, Maynooth University, W23 F2H6 Maynooth, Co, Kildare, Ireland; 7 Department of Physiology, Institute of Basic Medical Sciences, University of Oslo, Oslo, Norway; 8 Translational Health Sciences, St. Michael’s Hospital, Bristol Medical School, University of Bristol, Bristol, UK

## Abstract

**Background:**

Understanding the mechanisms of injury following neonatal hypoxic-ischemic encephalopathy (HIE) is a major goal in neonatal research. HIE can have severe effects on cognitive and motor development in newborns, including an increased risk of death. As the incidence is 10–20 times higher in low- and middle-income countries compared to developed countries, the interest in a therapy exists worldwide. Therapeutic hypothermia (HT) is the only effective treatment after HIE. However, TH is not universally effective, particularly in cases of inflammation-sensitized hypoxia-ischemia (HI); it provides limited benefit.

**Methods:**

To identify proteins that may contribute to the reduced efficacy of HT in the case of pre-HI inflammation sensitization, the proteomic profiles of animals subjected to HI and HT combined with lipopolysaccharide (LPS) were analyzed via liquid chromatography mass spectrometry (LC-MS/MS).

**Results:**

We identified proteins that potentially support the efficacy of HT and those that prevent the success of the therapy in the neonatal rat model of inflammation-sensitized HI.

**Conclusion:**

This study represents a step forward in identifying proteins related to the efficacy of HT following inflammation-sensitized HI.

**Impact:**

- Therapeutic hypothermia is the only available treatment for neonatal hypoxic-ischemic encephalopathy, but not effective in models of inflammation-sensitized hypoxic-ischemic brain injury.
- Using liquid chromatography mass spectrometry, we identified proteins possibly having an effect on the treatment success of therapeutic hypothermia following experimental inflammation-sensitized hypoxic-ischemic brain injury.
- This proteomic analysis reveals proteins as potential markers that could prevent or support the efficacy of therapeutic hypothermia in experimental neonatal inflammation-sensitized hypoxic-ischemic encephalopathy.

## Introduction

Hypoxic-ischemic encephalopathy (HIE) is a global risk factor for neonates, significantly impacting both the life span and quality of life of affected children. Depending on the severity of the insult, HIE can lead to neurodevelopmental disorders, impaired motor function, and neonatal death.^1–4^ As a global burden, it especially affects cases in low- and middle-income countries, as the incidence is 10–20 fold higher compared to developed countries.^5,6^ Therapeutic hypothermia (HT) is the only effective neuroprotective therapy that is currently available in developed countries. Within the first 6 h after birth, the newborn’s body temperature is reduced to 33–34 °C for 72 h. Nonetheless, HT is not beneficial in all cases of HIE.^7,8^ In cases that have been subjected to inflammatory stimuli before HIE, HT is less effective or not at all. This has been previously demonstrated experimentally by Osredkar et al.^9^ They displayed the effect of lipopolysaccharide (LPS) as a pre-inflammation agent in a newborn rat model of unilateral hypoxic-ischemic (HI) injury. Animals exposed to HI and HT had a significantly lower brain area loss compared to animals that underwent pre-injury inflammation with LPS injection. The brain damage found in these pre-HI inflammation-sensitized HI/HT animals showed no difference compared to the pre-HI inflammation-sensitized HI group without HT.^9^ This has also been observed in larger animal models.^10^

HT affects many pathways involved in the response to HI and can thereby influence both inflammatory and anti-inflammatory reactions in the newborn brain.^11^

To examine why HT is not effective in the pre-sensitized model, label-free liquid chromatography mass spectrometry (LC-MS/MS) was used to identify proteins that potentially support or prevent the efficacy of HT in the neonatal rat model of inflammation-sensitized HI.

Using LC-MS/MS, previous studies could reveal proteomic profiles of ischemia in rodents with HT treatment^12^ and comparing different time points after reperfusion.^13^ Yang et al. explored proteomic differences between HI and control groups in neonatal rats.^14^ Additional, uncovering the expression profiles of HIE in humans, recent studies published proteomic comparisons of HIE patients with healthy controls.^15,16^ These analyses uncovered hundreds of protein profiles with the potential for use as biomarkers and therapeutic targets. To direct the focus on the efficacy of HT in neonatal HIE subjected to inflammation before the insult, this paper uses proteomic analysis.

## Material and methods

### Animal model

**Fig. 1:**
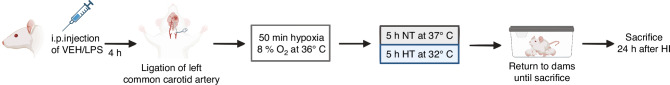
Experimental scheme of the animals used for LC-MS analysis. After LPS or Vehicle injection, animals underwent ligation of the left common carotid artery and hypoxic treatment, followed by either NT or HT treatment. After returning the animals to the dams, they were sacrificed 24 h afterthe HI insult. HI hypoxic ischemia, HT therapeutic hypothermia, i.p. intraperitoneal, LPS lipopolysaccharide, NT normothermia, VEH vehicle. Created with BioRender.com.

The brain hemispheres used for the proteomic analysis originated from previous experiments.^17^ Briefly, experiments were performed in 7-day-old (P7) Wistar rats (from Charles River, Sulzfeld, Germany). Animals were randomized across litter, sex, and weight. They were kept in an animal facility with conditions of a 12:12 light/dark cycle, a temperature of 19–21 °C, and food and water ad libitum. All experiments were approved by the University of Oslo’s Animal Ethics Research Committee. The experiment started with an intraperitoneal (i.p.) injection of either the vehicle solution (0.9% NaCl) or the LPS solution (*Escherichia coli* O55:B5, Sigma; 0.1 mg kg^−1^). Four hours after injection, the rats underwent the hypoxic-ischemic insult. Animals were anesthetized, and the left common carotid artery was ligated. Afterwards, the animals were exposed to 50 min hypoxia with 8% O_2_ at a rectal temperature (*T*_rec_) of 36 °C followed by either normothermia (NT) (5 h at *T*_rec_ 37 °C and 21% O_2_) or therapeutic hypothermia (HT) (5 h at *T*_rec_ 32 °C and 21% O_2_) treatment. According to the randomization there were four groups, each with *n* = 4 (Vehicle+HI/NT, LPS + HI/NT, Vehicle+HI/HT, and LPS + HI/HT) (see Table 1). The pups were returned to their dams until they were sacrificed. For this experiment, the animals were sacrificed 24 h after the HI insult (P8) (Fig. 1).

**Table 1:** Treatment groups.

i.p. injection	HI insult	Temperature treatment	Treatment group
Vehicle	HI	Normothermia	Veh+HI/NT
Vehicle	HI	Therapeutic Hypothermia	Veh+HI/HT
LPS	HI	Normothermia	LPS + HI/NT
LPS	HI	Therapeutic Hypothermia	LPS + HI/HT

### Sample preparation and mass spectrometry analysis

#### Materials

The proteomic analysis of formalin-fixed and paraffin-embedded (FFPE) brain specimens was performed using analytical grade chemicals from Bio-Rad Laboratories (Hemel-Hempstead, Hertfordshire, UK), GE Healthcare (Little Chalfont, Buckinghamshire, UK), and Sigma Chemical Company (Dorset, UK). Mass spectrometry-grade trypsin (MS-grade), spin filters (Vivacon 500, VN0H22; 30,000 MWCO), and protease inhibitors (cOmplete™, mini protease inhibitor cocktail) were purchased from ThermoFisher Scientific (Dublin, Ireland), Sartorius (Göttingen, Germany), and Roche (Mannheim, Germany), respectively. The protein concentration was determined using the Pierce 660 nm Protein Assay Reagent from ThermoFisher Scientific (Dublin, Ireland).

#### Label-free liquid chromatography mass spectrometry

The brain tissue was prepared and analyzed via label-free liquid chromatography mass spectrometry (LC-MS/MS). For the comparative survey of only hypoxia or LPS (right hemisphere) vs. hypoxia-ischemia treated (left hemisphere) brain tissue specimens, 32 samples were analyzed using 4 biological repeats of each analytical group using bottom-up proteomics. FFPE samples were deparaffinized, dehydrated, and lysed in extraction buffer (20 mM Tris-hydrochloric acid (HCl) buffer pH 9, containing 2% sodium dodecyl sulfate (SDS) with sonication and heating at 100 °C for 20 min. Protein concentrations were determined using the 660 nm Kit (ThermoFisher Scientific) following the manufacturer’s instructions, and as optimized for proteomic analyses.^18^ 10 µg of tissue lysates was mixed with 200 µL of urea buffer (8 M urea in 0.1 M Tris/HCl pH 8.5) and loaded onto 30 kDa Microcon filters (Sartorius-Vivacon 500). All on-column washing steps using the urea buffer were performed for 15 min at 14,000x*g*. After three washing steps (with 200 µL of urea buffer each time), proteins were reduced (100 μL 10 mM dithiothreitol (DTT) in 100 mM ammonium bicarbonate was added to the filter), vortexed, and incubated at 37 °C for 20 min.^19^ Following this, proteins were alkylated (100 μl 15 mM iodoacetamide (IAA) in 100 mM ammonium bicarbonate was added to the filter), vortexed, and incubated in the dark at room temperature for 20 min. Filters were centrifuged at 14,000 x *g* for 15 min and any remaining urea removed using two washing steps (with 200 μl of 100 mM ammonium bicarbonate). All on-column washing steps using the ammonium bicarbonate buffer were performed for 10 min at 14,000 x* g*. Samples were digested overnight at 37 °C using sequencing-grade trypsin (Promega) using a ratio of trypsin to protein of 1:50 (w:w) made up using 100 mM ammonium bicarbonate (40 μL of digestion buffer added to each filter).^20^ After the incubation time, filter units were transferred to new collection tubes and centrifuged at 14,000 x* g* for 10 min. 40 μl of 100 mM ammonium bicarbonate was subsequently added to each filter unit and centrifuged at 14,000 x *g* for 10 min. Samples were acidified (acidification buffer: 0.5% trifluoroacetic acid (TFA) in 20% acetonitrile (ACN)) using a ratio of 1 µL of acidification buffer for every 9 µL of sample. The mass spectrometric analysis of generated peptide populations was carried out by bottom-up proteomics^21^ using an optimized workflow, as previously described in detail.^22^ The LC-MS/MS analysis of peptides was performed with an Ultimate 3000 NanoLC system (Dionex Corporation, Sunnyvale, CA) coupled to a Q-Exactive mass spectrometer (ThermoFisher Scientific).

#### LC-MS/MS data analysis

For quantitative analysis, samples were evaluated using MaxQuant (v1.5.2.8) and the Andromeda search engine to identify the detected features against the UniProtKB/SwissProt database. The following search parameters were used: (i) first search peptide tolerance of 20 ppm, (ii) main search peptide tolerance of 4.5 ppm, (iii) cysteine carbamidomethylation set as a fixed modification, (iv) methionine oxidation set as a variable modification, (v) a maximum of two missed cleavage sites and (vi) a minimum peptide length of seven amino acids. The false discovery rate (FDR) was set to 1% for both peptides and proteins using a target-decoy approach. Proteins were quantified across samples using the label-free quantification algorithm (normalization) in MaxQuant as label-free quantification (LFQ) intensities. Perseus v.1.5.6.0 was used for data transformation, analysis, processing, and visualization. For systems bioinformatics analysis, the publicly available analysis tools PANTHER (http://www.pantherdb.org) and STRING (https://string-db.org) were employed for the identification of protein classes^23^ and potential protein-protein interaction patterns,^24^ respectively.

### Immunoflourescence staining

The FFPE brain specimens were cut into 2 µm coronal slices. The same samples with *n* = 4 were used as for the LC-MS/MS analysis. To remove the paraffin, the slices were incubated overnight at 60 °C and then incubated in xylene, followed by an alcohol series with decreasing concentrations for rehydration (5 min incubation time for each step; alcohol series: 100%, 80%, 70%, and 50% ethanol). After washing in phosphate-buffered saline (PBS), epitope retrieval was conducted by boiling the samples in citrate buffer (pH 6) for 5 min. To balance the pH of the samples, they were washed three times in PBS. To permeabilize the cell membrane, the slices were incubated in 0.1% Triton X-100 for 30 min. After blocking with 20% goat serum (diluted in PBS) for 30 min, the slices were incubated with the primary antibody anti-rabbit prosaposin (1:50) or anti-mouse triosephosphate isomerase (1:100) overnight at 4 °C (both purchased from Proteintech, diluted in 0.7% Carrageen solution). The next day, the slices were incubated with the secondary antibody goat-anti-rabbit IgG (H + L) Cross-Adsorbed Alexa Fluor™ 488 or goat-anti-mouse IgG (H + L) Cross-Adsorbed Alexa Fluor™ 594 (both 1:500 diluted in 0.7% Carrageen solution, purchased from Invitrogen) for 1 h in the dark. 4,6-diamidino-2-phenylindole (DAPI) was added via Fluoromount-G™ Mounting Medium, with DAPI (ThermoFisher). The slices were scanned with an AxioScan Z.1 microscope (Zeiss, Germany) and a confocal LSM800 microscope (Zeiss, Germany) and analyzed with ZEN Blue 3.1 (Carl Zeiss Microscopy GmbH). The intensity of the areas cortex, hippocampus and thalamus were measured and normalized to the area (µm^2^) (Figs. 4 and 5).

### Statistical analysis

The statistical analysis for the data of the LC-MS/MS was performed with the two-sample *t*-test with *p*-values of ≤0.05. The statistical analysis for the intensity measurement of the immunofluorescence staining was performed with the two-way ANOVA with *p*-values of <0.05.

## Results

### Proteomic profiles of analyzed groups

**Fig. 2:**
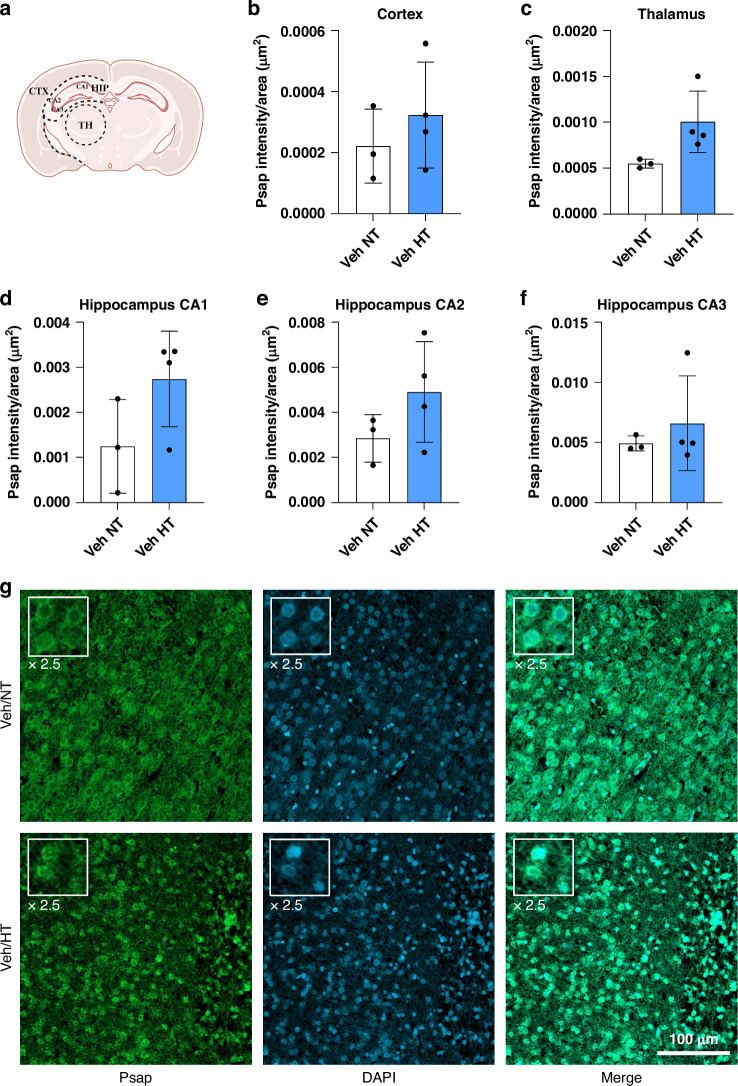
Intensity measurement of Psap in HI+Veh/HT (blue bar) compared to HI + Veh/NT (white bar). Analysis of Psap intensity per area (µm^2^) in cortex (**b**), thalamus (**c**) and hippocampus (divided into CA1-3, **d**–**f**). **a** Scheme of analyzed areas. Exemplary images of the staining show Psap staining in green and DAPI in blue (**g**). The insert is presenting a 2.5x magnification of exemplary cells. Localization: cortex. Scale bar: 100 µm. The two-way ANOVA test did not show any significant difference between the two treatment groups (*p*-value < 0.05). Scheme of analyzed areas created with BioRender.com.

Proteomic differences were revealed by MS analysis of the four groups. A total of 192 proteins were identified to be significantly increased or decreased when the groups were compared (Fig. 2). Comparing LPS + HI/HT with Veh+HI/NT showed the highest number of affected proteins (30 increased and 32 decreased proteins) and the HT treatment (Veh+HI/HT vs. Veh+HI/NT) presented the lowest number of affected proteins (23 increased and 22 decreased proteins). Treatment with LPS results in 29 significantly increased and 25 significantly decreased proteins in the hypothermia treatment compared to normothermia (LPS + HI/HT vs. LPS + HI/NT) (Fig. 2).

### Proteomic differences between positive and negative outcomes after therapeutic hypothermia

**Fig. 3:**
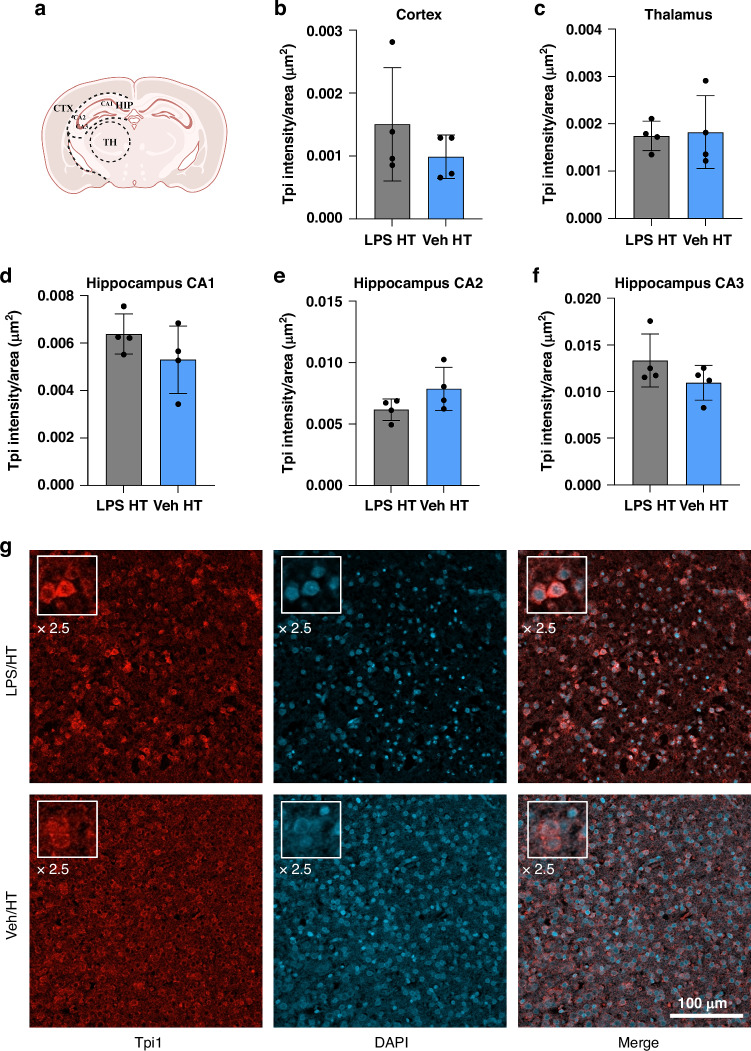
Intensity measurement of Tpi in HI + LPS/HT (gray bar) compared to HI + Veh/HT (blue bar). Analysis of Tpi intensity per area (µm^2^) in cortex (**b**), thalamus (**c**) and hippocampus (divided into CA1-3, **d**–**f**). **a** Scheme of analyzed areas. Exemplary images of the staining show Tpi staining in red and DAPI in blue (**g**). The insert is presenting a 2.5x magnification of exemplary cells. Localization: cortex. Scale bar: 100 µm. Two-way ANOVA did not show any significant difference between the two treatment groups (*p*-value < 0.05). Scheme of analyzed areas created with BioRender.com.

We focused on the Veh+HI/HT and LPS + HI/HT groups as they represent the relevant different outcomes after HT therapy at seven days after HI insult.^9^ Figure 3 and Table 2 present 60 proteins that were significantly increased or decreased in the LPS + HI/HT group compared to Veh+HI/HT. 36 increased proteins were found with fold changes from 1.14 (cAMP-dependent protein kinase type II-beta regulatory subunit (Prkar2b)) to 2.82 (Platelet-activating factor acetylhydrolase IB subunit alpha2 (Pafah1b2), Table 2). The highest fold-change of the 24 decreased proteins was −2.88, corresponding to mitochondrial fission 1 protein (Fis1), whereas the lowest fold-change was −1.07, corresponding to mitochondrial malate dehydrogenase (Mdh2).

**Table 2:** Differentially expressed proteins in LPS + HI/HT compared to Veh + HI/HT.

Protein	Gene	Peptides	Coverage %	Molecular mass (kDa)	LPS + HI/HT vs. Veh + HI/HT	
					*p*-value	Fold-change
Mitochondrial fission 1 protein	Fis1	4	33.6	17.008	0.0053664	−2.88
Platelet-activating factor acetylhydrolase IB subunit alpha2	Pafah1b2	5	41	25.581	0.00508927	2.82
Translocon-associated protein subunit alpha	Ssr1	2	8	32.065	0.0130373	2
NADH-cytochrome b5 reductase 3	Cyb5r3	6	31.6	34.127	0.0403759	1.74
Neurexin-1	Nrxn1	10	8.6	166.17	0.00524879	−1.7
Fructose-bisphosphate aldolase C	Aldoc	15	53.7	39.394	0.00110099	1.66
Guanine nucleotide-binding protein subunit beta-4	Gnb4	12	57.1	37.379	0.0109674	1.59
Synaptic vesicle membrane protein VAT-1	Vat1l	6	22.8	45.817	0.0493292	1.59
Proteasome subunit alpha type-2	Psma2	9	51.3	25.926	0.0370545	1.53
40S ribosomal protein S10	Rps10	5	29.1	18.916	0.0204732	1.52
40S ribosomal protein S15a	Rps15a	6	51.5	14.839	0.00120967	1.5
14-3-3 protein gamma	Ywhag	14	65.2	28.302	0.00551208	1.48
Glycogen phosphorylase, brain form	Pygb	29	40.2	96.729	0.0354713	1.48
6-phosphogluconate dehydrogenase, decarboxylating	Pgd	6	23	53.247	0.00692635	1.47
Serine/threonine-protein phosphatase 2 A 55 kDa regulatory subunit B alpha isoform	Ppp2r2a	10	34.2	51.691	0.0130427	−1.44
Proteasome subunit beta type-3	Psmb3	5	30.2	22.965	0.0185851	1.42
ADP-ribosylation factor-like protein 8 A	Arl8a	10	74.2	21.39	0.00974207	−1.42
Actin-related protein 2/3 complex subunit 3	Arpc3	4	32	20.524	0.04952	1.41
Protein arginine N-methyltransferase 5	Prmt5	10	20.6	72.679	0.0207775	1.41
Prosaposin	Psap	6	12	61.422	0.00770054	−1.41
Transcriptional activator protein Pur-beta	Purb	4	24.7	33.901	0.0151871	1.37
Calnexin	Canx	9	19.8	67.277	0.00688106	−1.37
Alpha-tubulin N-acetyltransferase 1	Atat1	11	36.1	47.163	0.042311	−1.33
40S ribosomal protein S3a	Rps3a	13	46.2	29.885	0.00500285	1.31
Neurobeachin	Nbea	15	7	326.74	0.0123096	−1.31
Peroxiredoxin-2	Prdx2	7	53	21.778	0.00349144	1.3
Triosephosphate isomerase	Tpi1	7	42.2	26.712	0.004595	1.27
Synaptobrevin	Ykt6	7	43.4	22.314	0.00576037	−1.27
Tenascin	Tnc	37	25.9	231.8	0.0225137	−1.27
Malate dehydrogenase, cytoplasmic	Mdh1	9	33.5	36.511	0.00300204	1.26
Glycogen synthase kinase-3 alpha	Gsk3a	7	23.1	51.66	0.0348215	1.25
Leucine-rich repeat-containing protein 47	Lrrc47	7	16.2	63.589	0.0479829	1.25
Fatty acid synthase	Fasn	36	20.4	272.43	0.0303762	-1.25
Phosphoglycerate mutase 1	Pgam1	12	65.7	28.832	0.0154497	1.24
Phosphoribosyl pyrophosphate synthase-associated protein 2	Prpsap2	12	47.7	40.88	0.00560947	1.24
Histone-binding protein RBBP4	Rbbp4	7	21.9	47.655	0.015914	−1.24
ADP-ribosylation factor 5	Arf5	11	74.4	20.529	0.0079629	1.23
Asparagine--tRNA ligase, cytoplasmic	NARS1	16	32.6	64.279	0.0145767	−1.23
Guanine nucleotide-binding protein G(s) subunit alpha isoforms short	Gnas	13	39.1	45.663	0.0285632	−1.23
Endophilin-B1	Sh3glb1	4	18.1	40.855	0.034113	1.22
Proteasome subunit alpha type-5	Psma5	8	48.5	26.411	0.0237352	1.21
Elongation factor 2	Eef2	35	52.6	95.313	0.00533489	−1.21
Eukaryotic translation initiation factor 5A-1	Eif5a	10	77.9	16.832	0.00613205	−1.21
Calretinin	Calb2	12	53.5	31.372	0.00755759	1.2
Creatine kinase U-type, mitochondrial	Ckmt1	12	39.5	47.003	0.0493006	1.2
Guanine nucleotide-binding protein G(i) subunit alpha	Gnai3	13	46.3	40.538	0.0312879	−1.2
Protein RUFY3	Rufy3	12	32.2	53.006	0.0296503	−1.19
Cytosolic acyl coenzyme A thioester hydrolase	Acot7	9	36.2	42.536	0.0341218	1.18
AP-2 complex subunit sigma	Ap2s1	6	44.4	17.018	0.0171208	1.17
Creatine kinase B-type	Ckb	18	70.9	42.713	0.0168094	1.17
Protein rogdi homolog	Rogdi	5	21.6	32.1	0.0361759	1.17
Aspartyl aminopeptidase	Dnpep	12	45.2	52.206	0.0192393	1.16
Citrate synthase, mitochondrial	Cs	11	24.1	51.736	0.0042455	−1.16
cAMP-dependent protein kinase type II-beta regulatory subunit	Prkar2b	19	60.3	46.167	0.0449576	1.14
Voltage-dependent anion-selective channel protein 2	Vdac2	11	49.5	31.732	0.0351051	−1.14
F-actin-capping protein subunit alpha-2	Capza2	12	67.5	32.967	0.0164711	1.12
Cytochrome b-c1 complex subunit 1, mitochondrial	Uqcrc1	9	23.3	52.851	0.0418003	−1.11
Glyceraldehyde-3-phosphate dehydrogenase	Gapdh	17	61.9	35.81	0.0343714	−1.11
Transforming protein RhoA	Rhoa	7	56	21.782	0.0445361	−1.1
Malate dehydrogenase, mitochondrial	Mdh2	15	57.7	35.611	0.0256889	−1.07

*HT* therapeutic hypothermia, *LPS* lipopolysaccharide, *Veh* vehicle.

A two-sample *t*-test (*p*-values of ≤0.05) was used.

Functional protein-protein interactions of regulated proteins were presented using STRING analysis (Fig. 3). STRING analysis predicted strong interactions between glyceraldehyde-3-phosphate dehydrogenase (Gapdh), citrate synthase (Cs), triosephosphate isomerase (Tpi1) and malate dehydrogenase 1 and 2 (Mdh1 and Mdh2).

The Kyoto Encyclopedia of Genes and Genomes (KEGG) analysis identified 13 functional pathways related to significantly increased protein expression and 29 related to significantly decreased protein expression in LPS + HI/HT compared to Veh+HI/HT (Supplementary Fig. S1). The highest number of proteins was related to metabolic pathways (11 increased proteins). Gene ontology analysis showed that a variety of processes were influenced when HT was performed with LPS pre-HI sensitization. Biological processes, molecular functions, and cellular components were involved in the different protein expression patterns (Supplementary Fig. S2).

### Proteins possibly supporting the effect of therapeutic hypothermia

We focused our analysis on proteins potentially involved in the neuroprotective effects of HT. In the Veh + HI/HT group compared to Veh + HI/NT, five proteins were uniquely significantly increased when concomitantly significantly decreased in LPS + HI/HT compared to Veh+HI/HT; such as histone-binding protein (RBBP4), Mdh2, prosaposin (Psap), RUN, and FYVE domain-containing 3 (Rufy3) and voltage-dependent anion-selective channel protein 2 (Vdac2) (Fig. 3b, c and Table 3). STRING analysis did not predict potential or known connections among Psap, RBP4, and Rufy3. Mdh2 and Vdac2 were connected by co-expression and text mining. The protein with the highest fold changes in protein expression between the compared groups was Psap with values of −1.41 (LPS + HI/HT vs. Veh+HI/HT) and 1.48 (Veh + HI/HT vs. Veh + HI/NT) (Table 3).

**Table 3:** Identified proteins increased in the Veh + HI/HT group vs. Veh + HI/NT, and decreased in the LPS + HI/HT group vs. Veh + HI/HT. A two-sample *t*-test (*p*-values of ≤0.05) was used.

Protein	Gene	Peptides	Coverage %	Molecular mass (kDa)	Veh + HI/HT vs. Veh + HI/NT		LPS + HI/HT vs. Veh + HI/HT	
					*p*-value	Fold-change	*p*-value	Fold-change
Histone-binding protein RBBP4	Rbbp4	7	21.9	47.655	0.0426667	1.11	0.015914	−1.24
Malate dehydrogenase, mitochondrial	Mdh2	15	57.7	35.611	0.0426667	1.11	0.0256889	−1.07
Prosaposin	Psap	6	12	61.422	0.0345421	1.48	0.00770054	−1.41
Protein RUFY3	Rufy3	12	32.2	53.006	0.0418226	1.21	0.0296503	−1.19
Voltage-dependent anion-selective channel protein 2	Vdac2	11	49.5	31.732	0.0155103	1.17	0.0351051	−1.14

Gene ontology analysis revealed that two of the five proteins, Psap and Vdac2, were related to ceramide binding (Supplementary Table S1). KEGG analysis did not show any relation.

**Fig. 4:**
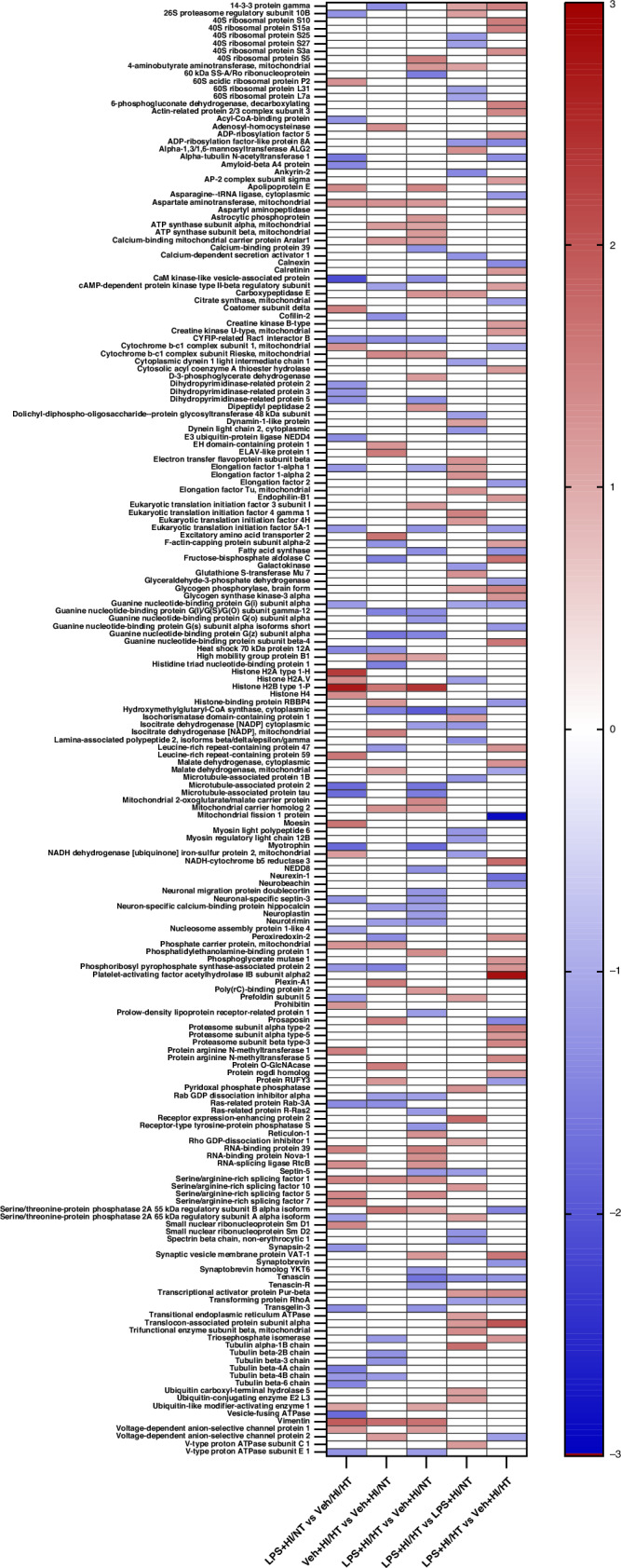
Proteomic profiles of experimental groups compared to each other. Heat map of regulated proteins based on fold-change. Increased protein expression is presented in red, and decreased proteins are presented in blue. A two-sample *t*-test (*p*-values of ≤0.05) was used to identify the proteins.

Immunofluorescence staining was performed with Psap as a representative of the five proteins. As presented in Fig. 4, Psap was more highly expressed in the Veh+HI/HT group compared to Veh + HI/NT in all analyzed areas. The intensity measurements showed the same trend as presented by the proteomic results.

### Proteins possibly preventing the effect of therapeutic hypothermia

Six proteins were identified as potential factors that inhibit the neuroprotective effects of HT because of their unique significant decrease in the Veh+HI/HT group compared to Veh+HI/NT and their significant increase in the LPS + HI/HT group compared to Veh+HI/HT: Prkar2b, F-actin-capping protein subunit alpha-2 (Capza2), fructose-bisphosphate aldolase C (ALDOC), leucine-rich repeat-containing protein 47 (Lrrc47), peroxiredoxin-2 (Prdx2) and Tpi1 (Fig. 3d, e and Table 4).

**Table 4:** Identified proteins decreased in the Veh + HI/HT group vs.

Protein	Gene	Peptides	Coverage %	Molecular mass (kDa)	Veh + HI/HT vs. Veh + HI/NT		LPS + HI/HT vs. Veh + HI/HT	
					*p*-value	Fold-change	*p*-value	Fold-change
cAMP-dependent protein kinase type II-beta regulatory subunit	Prkar2b	19	60.3	46.167	0.0495396	−1.11	0.0449576	1.14
F-actin-capping protein subunit alpha-2	Capza2	12	67.5	32.967	0.0246418	−1.28	0.0164711	1.12
Fructose-bisphosphate aldolase C	Aldoc	15	53.7	39.394	0.0060543	−1.49	0.00110099	1.66
Leucine-rich repeat-containing protein 47	Lrrc47	7	16.2	63.589	0.0322275	−1.22	0.0479829	1.25
Peroxiredoxin-2	Prdx2	7	53	21.778	0.00579937	−1.36	0.00349144	1.3
Triosephosphate isomerase	Tpi1	7	42.2	26.712	0.0464166	−1.21	0.004595	1.27

Veh + HI/NT and increased in the LPS + HI/HT group vs. Veh + HI/HT. A two-sample *t*-test (*p*-values of ≤ 0.05) was used.

ALDOC showed the highest increase in the LPS + HI/HT group compared to Veh+HI/HT (fold-change of 1.66) and the highest decrease in the Veh+HI/HT group compared to Veh + HI/NT (fold-change of −1.49). STRING analysis revealed ALDOC interaction and co-expression with Tpi1 (Fig. 3d). Tpi1 was concomitantly associated with Prdx2 (Fig. 3d).

KEGG analysis revealed that two of the six proteins, ALDOC and Tpi1, were related to fructose and mannose metabolism, glycolysis/gluconeogenesis, biosynthesis of amino acids, and carbon metabolism (Supplementary Table S2). The gene ontology analysis did not show any relation between these six proteins.

**Fig. 5:**
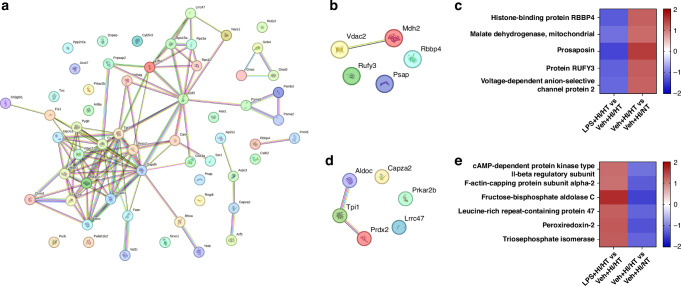
Proteomic differences between treatment groups. **a** Differentially expressed proteins in LPS + HI/HT compared to Veh+HI/HT. STRING network of regulated proteins. The number of nodes was 58, the average node degree was 3.69. **b**,** c** Proteins increased in Veh+HI/HT vs. Veh+HI/NT and decreased in LPS + HI/HT vs. Veh+HI/HT. **b** STRING network of regulated proteins. The number of nodes was 5, the average node degree was 0.4. **c** Heat map of regulated proteins based on fold-change. **d**,** e** Proteins decreased in Veh+HI/HT vs. Veh+HI/NT and increased in LPS + HI/HT vs. Veh+HI/HT. **d** STRING network of regulated proteins. The number of nodes was 6, the average node degree was 0.667. **e** Heat map of regulated proteins based on fold-change. A two-sample *t*-test (*p*-values of ≤0.05) was used to identify the proteins. Legend for STRING networks: Known interactions from curated databases (turquoise), known interactions experimentally determined (pink), predicted interactions via gene neighborhood (dark green), predicted interactions via gene fusions (red), predicted interactions via gene-occurrence (dark blue), text mining (light green), co-expression (black), protein homology (light blue) (string-db.org).

Representing these six proteins, an immunofluorescence staining was performed with Tpi1 (Fig. 5). The staining showed differences between the LPS + HI/HT and Veh+HI/HT groups. In the LPS + HI/HT group, Tpi1 was expressed more strongly in the areas cortex, hippocampal CA1, and CA3 than in the Veh+HI/HT group. The staining of these areas confirmed the proteomic data (Fig. 5).

## Discussion

This study focused on identifying proteins that might explain why HT may be ineffective in cases of HIE sensitized with LPS. Pre-HI sensitization with LPS leads to inflammatory reactions and is a model for inflammation-sensitized HI brain injury.^9^ Mass spectrometry enabled us to retrace the proteomic profiles of brain hemispheres from animals following HI. We have found that proteomic analysis revealed proteins that could potentially be used to describe the efficacy of HT in the setting of pre-HI inflammation sensitization and HIE.

By comparing the two HT treatment groups, Veh+HI/HT and LPS + HI/HT, we found a difference in the expression of 60 proteins, which could have an effect on different outcomes after HT. Pafah1b2 showed the highest increase in LPS + HI/HT compared to Veh+HI/HT, with a likely contribution to poor outcomes after cooling therapy. The phospholipase Pafah1b2 was found to be upregulated after traumatic brain injury.^25^ It is connected to amyloid-β secretion as its knockdown can reduce the amount of amyloid-β, which is linked to Alzheimer’s Disease.^26^ The expression of Pafah1b2 was discovered to be mediated by HIF1a.^27^ As there is no published data about the effects of Pafah1b2 on brain injury after HI, this aspect warrants further investigation.

The level of Fis1 was significantly decreased in the Veh+HI/HT group compared to LPS + HI/HT. Fis1 is involved in mitochondrial fission, mitophagy and the general mitochondrial dynamics.^28^ Its regulation determines the mitochondrial condition as its overexpression can lead to mitochondrial fragmentation, increased ROS, while its knockdown promotes mitochondrial elongation, decreased ROS, decreased mitophagy and mitochondrial respiration.^28^ Its protein abundance could influence the outcome after HIE and thereby serve as a marker for dysregulated cell metabolism.

The various interactions between the significantly increased and decreased proteins reveal the complexity underlying the reactions to HI and HT. To specify the analysis, we directed the study towards the question of the efficacy of HT. Six proteins were hypothesized to negatively affect the outcome after pre-HI inflammation sensitization and HT treatment. As shown by Osredkar et al., HT had no protective effect following HI when pre-HI sensitized with LPS. The percentage of brain area loss in pre-HI sensitized animals that received HT was the same as in animals that did not receive HT. In contrast, in animals that were not pre-HI sensitized, HT improved the outcome after the HI insult, with reduced tissue loss and reduction in mortality.^7,9^

Tpi1 is induced by hypoxia on the mRNA and protein level.^29,30^ Tpi1 is regulated via the HIF pathway and thereby a part of oxygen-regulated proteins.^29^ Its hypoxic upregulation promotes the shift from the oxidative phosphorylation to the anaerobic glycolysis.^29,30^ The regulation of Tpi1 is crucial as it is connected to the production of a neurotoxic substrate and to Alzheimer’s disease.^31^

Tpi1 is post-translationally modifiable and thus could potentially be targeted.^32^ The activity of Tpi1 is partly dependent on the activity of ALDOC, a protein that also plays a critical role in the outcome of HT. ALDOC cleaves hexose and produces glyceraldehyde-3-phosphate (G-3-P) and dihydroxy-acetone-phosphate (DHAP). Tpi1 further converts DHAP into G-3-P, and is thereby affected by ALDOC activity.^29^

ALDOC is also oxygen-regulated and regulated by the HIF pathway.^29^ In a rat model of controlled cortical impact, Thelin et al. showed the involvement of ALDOC in hypoxic regulation and signaling. ALDOC was detected early after the injury and significantly decreased over time.^33^

It is involved in the progress of HIE, as it was detected to be significantly increased in infants with adverse outcomes after HIE compared to healthy controls.^16^ Thus, ALDOC could potentially serve as a marker and therapeutic target.

It is unclear on which level ALDOC could impair the molecular mechanisms following HT. Chen et al. deduced from their results that ALDOC might impact the polarization and thereby the function of macrophages.^34^ A mutation in ALDOC has been shown to increase the number of macrophages with a pro-inflammatory phenotype and decrease the number of anti-inflammatory macrophages.^34^ In gastric cancer cells it could be discovered that ALDOC lead to the opposite trend of macrophage polarization but supporting tumor initiation and progression.^35^ The authors concluded from their data that the HIF pathway plays a critical role in macrophage polarization. As ALDOC is regulated by the HIF pathway, ALDOC might impact the macrophage polarization via HIF.^29,34,35^

In our model of neonatal HIE in the context of HT treatment, ALDOC could prevent the efficacy of HT by supporting an inflammatory phenotype of macrophages and thereby increasing inflammation in the brain.

Our proteomic findings and the resulting hypothesis that Tpi1 and ALDOC could prevent successful HT treatment are compatible with the existing research on the two proteins.

Peroxiredoxin (Prdx2) is a protein known for its anti-oxidative activities. It neutralizes reactive oxygen species (ROS) and protects the cells.^36–39^ Intracellular Prdx2 seems to be neuroprotective, whereas extracellular Prdx2, mostly released by necrotic cells, can contribute to inflammation and lead to cell death.^36^ Prdx2 shows increased levels in neurodegenerative diseases^37,39^ and after ischemic stress.^36^ The significant increase of Prdx2, which was also visible in our data in the LPS + HI/HT group, could represent Prdx2 supporting inflammation and further brain damage. Further investigation is necessary to elucidate whether Prdx2 acts neuroprotectively, proinflammatorily, or both to understand its role in the case of HI.

Five proteins were identified that possibly have a positive impact on HT after HI. Psap was shown to be significantly increased in the treatment group when HT was effective 7 days after HI regarding brain area loss and significantly decreased when it was ineffective at this time point.^9^ The contribution of Psap to the positive outcome of therapy can be explained by its role in neuroprotection. Psap is a glycoprotein and lysosomal precursor of saposins A–D.^40–42^ It has been defined as a neurotrophic factor since O’Brien et al. showed its promoting role in neurite outgrowth.^40^ Psap was also reported to act neuroprotective in a gerbil model with ischemic treatment. Sano et al. presented a protective effect of Psap on the hippocampal CA1 neurons and the prevention of learning disability due to ischemia.^43^ Another study showed a disruptive effect of cerebral ischemia on Psap processing and glycosphingolipid metabolism.^42^ Cerebral ischemia in mice leads to upregulation of Psap within the hippocampus owing to altered lysosomal Psap processing. The authors ended their discussion of the results with the question of whether alteration of Psap processing acts neuroprotectively or if it represents just an event following lysosomal disruption. Our results showed a significant increase in Psap in a successful therapy and a significant Psap decrease in the opposite outcome; the Psap processing might be neuroprotective. This hypothesis is supported by Fujita et al., who demonstrated that Psap-deficiency in mice leads to hypomyelination, neurological impairments, and a shortened life span.^44^

Rufy could contribute to a positive outcome after HIE is treated with HT. Its possible protective impact has already been demonstrated in a rat model of brain damage after subarachnoid hemorrhage by Wang et al. Rufy3 overexpression decreased brain damage and Rufy3 knock-out aggravated neurological deficits in tested animals.^45^ Rufy3 interacts with fascin to change the actin filament arrangement, and thereby, the morphogenesis of neuronal axons. The protein increases axon length and promotes axon repair and synaptic plasticity. Thereby, it protects from brain damage by inhibiting axonal injury.^45,46^ The impact of HI and HT on Rufy3 requires further investigation to determine if it specifically reacts to this stimulus and in which cases it is acting neuroprotective.

### Limitations

This study presents an analysis of proteomic data, and the results are reliable and valid. However, there are limitations to our study. Our hypothesis could be confirmed by immunofluorescence staining of two representative proteins. Nonetheless, this only validates a part of the dataset. The validation is limited as there was no significant difference between the groups. After LC-MS/MS, it is not known if the significantly increased and decreased proteins are cause or effect of the treatment. These proteins could influence the efficacy of HT, or they may be differently affected by it.

As this study only focuses on one question regarding the efficacy of HT, there are still other possible questions to be answered with the presented dataset. To reveal the general mechanism of HT, a future study could compare the proteomic profile of HT against NT after HI injury without the pre-HI inflammation sensitization with LPS.

The comparison with sham animals was not a part of this experiment and was therefore missing. This represents a major limitation to this study. These experiments need to be performed in future studies. This paper analyzes samples originating from a previous experiment^17^ and hypotheses within are partially based on the analysis of tissue injury analyzed 7 days after HI by Osredkar et al.^9^ There is no proof of brain injury grade for the 24 h time point. Nonetheless, the findings made at the 24 h time point could validate the previous brain injury results at a cellular level regarding neuronal death, apoptosis, astrogliosis, and microglial activation.^9,17^ Another limitation is the lack of comparison between sexes. As four animals from each group were pooled for LC-MS/MS, it was not possible to analyze the influence of sex. To correspond to the LC-MS/MS analysis, the intensity measurement does not include sex comparison either. Blood analysis would be more effective for the general discovery of predictive markers. This was not conducted in this experiment, as the analysis focused on the tissue samples. The fold-change values between the groups were small, which restricts the analysis of specific markers.

## Conclusion

Our proteomic analysis revealed proteins that could potentially be used to describe the efficacy of HT in the setting of HIE and inflammation. Proteins that were mentioned as neuroprotective in previous publications were found to be significantly increased in animals with successful HT treatment and significantly decreased in animals with ineffective HT. Proteins with potentially damaging characteristics showed the opposite trend. More research is necessary to further validate Psap and Rufy3 as attributes supporting, and Tpi1 and ALDOC as attributes preventing the success of HT after LPS-sensitized HI.

## Supplementary information

Supplementary information

## Data Availability

The raw data supporting the conclusions of this article will be made available by the authors, without undue reservation, to any qualified researcher.
