# Supplementary material for: Proteomic analysis identifying proteins relevant for treatment success following experimental neonatal inflammation-sensitized hypoxia-ischemia
Source: Pediatr Res. 2025 May 13;99(4):1591–601. doi: 10.1038/s41390-025-04097-8 (PMC13102700; doi:10.1038/s41390-025-04097-8)
Supplement: Supplementary file 1 — Supplementary information [file 41390_2025_4097_MOESM1_ESM.pdf]

Supplementary Material

Supplementary Table S1: Gene Ontology (GO) Analysis of proteins increased in the Veh+HI/HT group vs Veh+HI/NT and decreased in the LPS+HI/HT group vs Veh+HI/HT. A two-sample t-test (p-values of ≤ 0.05) was used to identify the proteins.

**Protein**		**GO Analysis**
Prosaposin	Psap	Molecular function: Ceramide binding
Histone-binding protein RBBP4	Rbbp4	-
Protein RUFY3	Rufy3	-
Voltage-dependent anion-selective channel protein 2	Vdac2	Molecular function: Ceramide binding
Malate dehydrogenase, mitochondrial	Mdh2	-

Supplementary Table S2: KEGG Analysis of proteins decreased in the Veh+HI/HT group vs Veh+HI/NT and increased in the LPS+HI/HT group vs Veh+HI/HT. A two-sample t-test (p-values of ≤ 0.05) was used to identify the proteins. KEGG = Kyoto Encyclopedia of Genes and Genomes.

**Protein**		**KEGG Analysis**
Fructose-bisphosphate aldolase C	Aldoc	Fructose and mannose metabolism<br>Glycolysis / Gluconeogenesis<br>Biosynthesis of amino acids<br>Carbon metabolism
Peroxiredoxin-2	Prdx2	-
Triosephosphate isomerase	Tpi1	Fructose and mannose metabolism<br>Glycolysis / Gluconeogenesis<br>Biosynthesis of amino acids<br>Carbon metabolism
Leucine-rich repeat-containing protein 47	Lrrc47	-
cAMP-dependent protein kinase type II-beta regulatory subunit	Prkar2b	-
F-actin-capping protein subunit alpha-2	Capza2	-

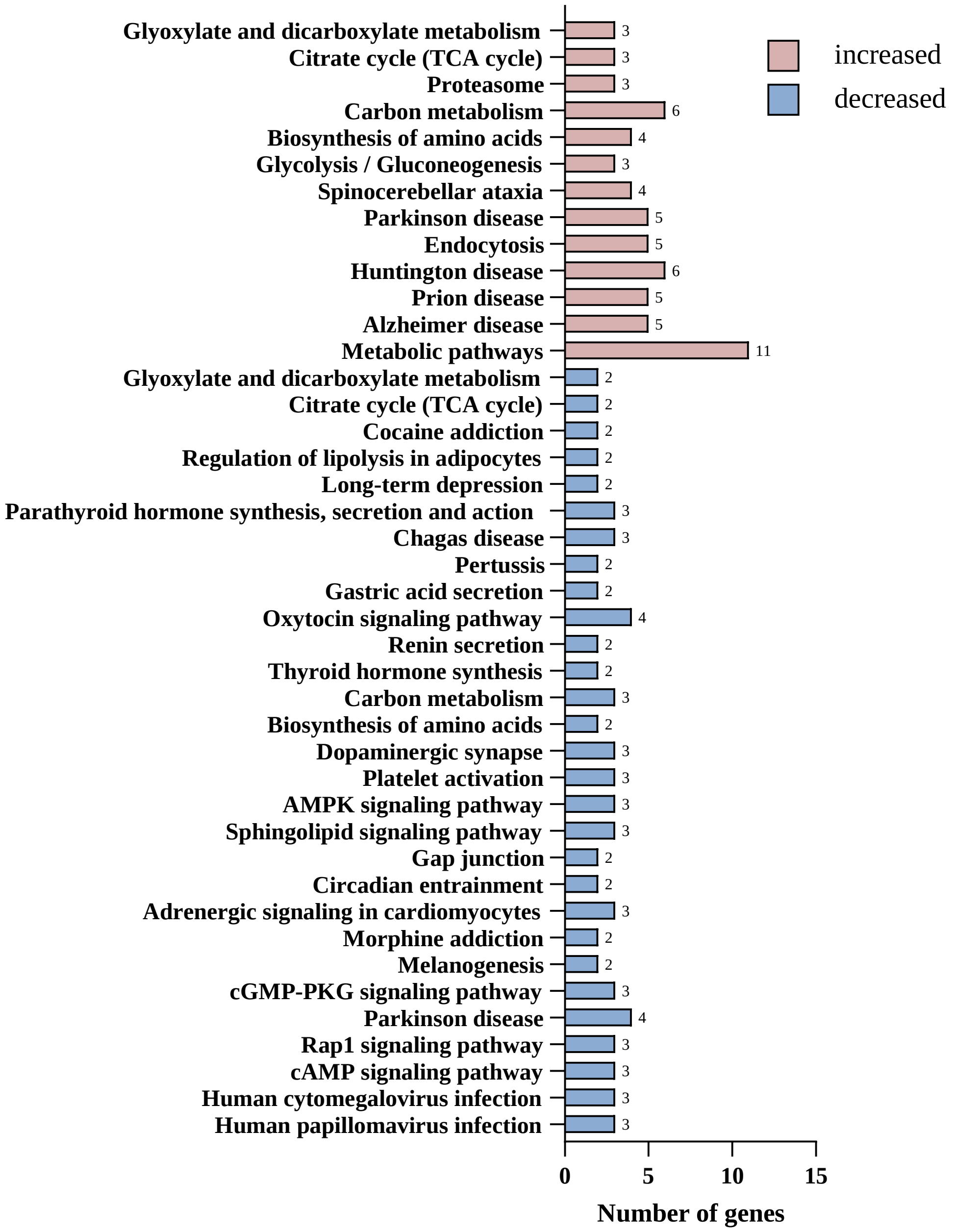

increased
decreased
Glyoxylate and dicarboxylate metabolism 3
Citrate cycle (TCA cycle) 3
Proteasome 3
Carbon metabolism 6
Biosynthesis of amino acids 4
Glycolysis / Gluconeogenesis 3
Spinocerebellar ataxia 4
Parkinson disease 5
Endocytosis 5
Huntington disease 6
Prion disease 5
Alzheimer disease 5
Metabolic pathways 11
Glyoxylate and dicarboxylate metabolism 2
Citrate cycle (TCA cycle) 2
Cocaine addiction 2
Regulation of lipolysis in adipocytes 2
Long-term depression 2
Parathyroid hormone synthesis, secretion and action 3
Chagas disease 3
Pertussis 2
Gastric acid secretion 2
Oxytocin signaling pathway 4
Renin secretion 2
Thyroid hormone synthesis 2
Carbon metabolism 3
Biosynthesis of amino acids 2
Dopaminergic synapse 3
Platelet activation 3
AMPK signaling pathway 3
Sphingolipid signaling pathway 3
Gap junction 2
Circadian entrainment 2
Adrenergic signaling in cardiomyocytes 3
Morphine addiction 2
Melanogenesis 2
cGMP-PKG signaling pathway 3
Parkinson disease 4
Rap1 signaling pathway 3
cAMP signaling pathway 3
Human cytomegalovirus infection 3
Human papillomavirus infection 3
0
5
10
15
Number of genes

Supplementary Figure S1: KEGG Analysis of increased (red) and decreased (blue) proteins in LPS+HI/HT compared to Veh+HI/HT. Numbers represent the amount of genes (analyzed with string-db.org). A two-sample t-test (p-values of ≤ 0.05) was used to identify the proteins. KEGG = Kyoto Encyclopedia of Genes and Genomes.

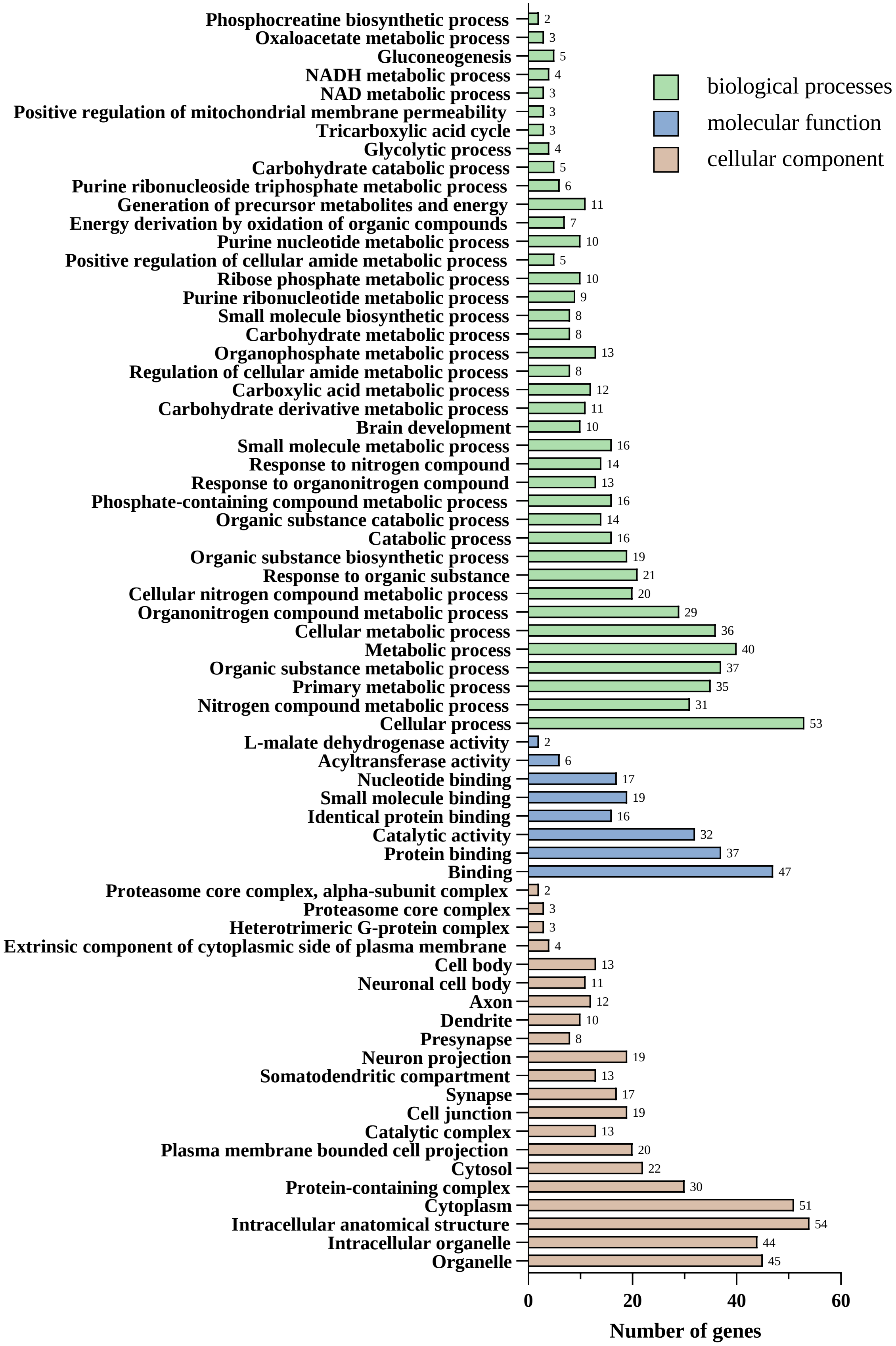

biological processes
molecular function
cellular component
Phosphocreatine biosynthetic process 2
Oxaloacetate metabolic process 3
Gluconeogenesis 5
NADH metabolic process 4
NAD metabolic process 3
Positive regulation of mitochondrial membrane permeability 3
Tricarboxylic acid cycle 3
Glycolytic process 4
Carbohydrate catabolic process 5
Purine ribonucleoside triphosphate metabolic process 6
Generation of precursor metabolites and energy 11
Energy derivation by oxidation of organic compounds 7
Purine nucleotide metabolic process 10
Positive regulation of cellular amide metabolic process 5
Ribose phosphate metabolic process 10
Purine ribonucleotide metabolic process 9
Small molecule biosynthetic process 8
Carbohydrate metabolic process 8
Organophosphate metabolic process 13
Regulation of cellular amide metabolic process 8
Carboxylic acid metabolic process 12
Carbohydrate derivative metabolic process 11
Brain development 10
Small molecule metabolic process 16
Response to nitrogen compound 14
Response to organonitrogen compound 13
Phosphate-containing compound metabolic process 16
Organic substance catabolic process 14
Catabolic process 16
Organic substance biosynthetic process 19
Response to organic substance 21
Cellular nitrogen compound metabolic process 20
Organonitrogen compound metabolic process 29
Cellular metabolic process 36
Metabolic process 40
Organic substance metabolic process 37
Primary metabolic process 35
Nitrogen compound metabolic process 31
Cellular process 53
L-malate dehydrogenase activity 2
Acyltransferase activity 6
Nucleotide binding 17
Small molecule binding 19
Identical protein binding 16
Catalytic activity 32
Protein binding 37
Binding 47
Proteasome core complex, alpha-subunit complex 2
Proteasome core complex 3
Heterotrimeric G-protein complex 3
Extrinsic component of cytoplasmic side of plasma membrane 4
Cell body 13
Neuronal cell body 11
Axon 12
Dendrite 10
Presynapse 8
Neuron projection 19
Somatodendritic compartment 13
Synapse 17
Cell junction 19
Catalytic complex 13
Plasma membrane bounded cell projection 20
Cytosol 22
Protein-containing complex 30
Cytoplasm 51
Intracellular anatomical structure 54
Intracellular organelle 44
Organelle 45
0
20
40
60
Number of genes

Supplementary Figure S2: Gene Ontology Analysis of genes with changed protein expression in LPS+HI/HT compared to Veh+HI/HT. Processes are divided into biological processes (green), molecular function (blue) and cellular component (orange). Numbers represent the amount of genes (analyzed with string-db.org). A two-sample t-test (p-values of ≤ 0.05) was used to identify the proteins.
